# Ultra-Low-Power FinFETs-Based TPCA-PUF Circuit for Secure IoT Devices

**DOI:** 10.3390/s21248302

**Published:** 2021-12-11

**Authors:** Cancio Monteiro, Yasuhiro Takahashi

**Affiliations:** 1 Department of Electronics and Electrical Engineering (EEE), Faculty of Engineering, Science and Technology, Universidade Nacional Timor Lorosa’e (UNTL), Avenida Hera, Cristo-Rei, Dili 314, Timor-Leste; 2 Department of Electrical, Electronic and Computer Engineering, Faculty of Engineering, Gifu University, 1-1 Yanagido, Gifu-shi 501-1193, Japan; yasut@gifu-u.ac.jp

**Keywords:** physical unclonable function (PUF), secure keys, FinFETs, low power, adiabatic, reliable and unique identity, IoT security, SRAM PUF

## Abstract

Low-power and secure crypto-devices are in crucial demand for the current emerging technology of the Internet of Things (IoT). In nanometer CMOS technology, the static and dynamic power consumptions are in a very critical challenge. Therefore, the FinFETs is an alternative technology due to its superior attributes of non-leakage power, intra-die variability, low-voltage operation, and lower retention voltage of SRAMs. In this study, our previous work on CMOS two-phase clocking adiabatic physical unclonable function (TPCA-PUF) is evaluated in a FinFET device with a 4-bits PUF circuit complexity. The TPCA-PUF-based shorted-gate (SG) and independent-gate (IG) modes of FinFETs are investigated under various ambient temperatures, process variations, and ±20% of supply voltage variations. To validate the proposed TPCA-PUF circuit, the QUALPFU-based Fin-FETs are compared in terms of cyclical energy dissipation, the security metrics of the uniqueness, the reliability, and the bit-error-rate (BER). The proposed TPCA-PUF is simulated using 45 nm process technology with a supply voltage of 1 V. The uniqueness, reliability, and the BER of the proposed TPCA-PUF are 50.13%, 99.57%, and 0.43%, respectively. In addition, it requires a start-up power of 18.32 nW and consumes energy of 2.3 fJ/bit/cycle at the reference temperature of 27 °C.

## 1. Introduction

**Figure 1:**
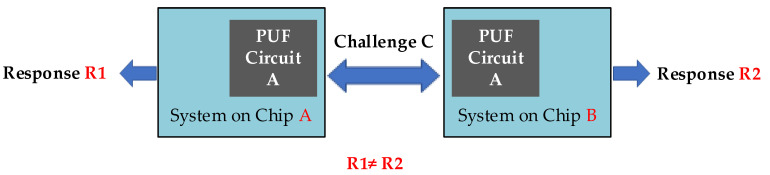
Two identical PUF circuits on two different chips generate different responses (modified from [15]).

In recent years, the emerging Internet of Things (IoT) technology has simultaneously introduced challenges and opportunities for engineering-related fields. IoT devices include smartphones, smart cards, biomedical devices, radio frequency identification (RFID) tags, and many other cryptographic devices that require security characteristics, such as authenticity, integrity, and confidentiality in performing their tasks [1,2]. Security is one of the main current challenges for IoT [3,4,5,6] as it begins to materialize in our daily life and future industrial systems (Industry 4.0) [7]. IoT devices must face several tough challenges, such as low energy consumption, lack of computational resources [8,9], as well as the need to secure devices against cyber-attacks [10]. Authentication, authorization, and privacy are three sides of the security triangle in IoT. Authentication is the first barrier in front of cyber-attacks. Physical unclonable functions (PUFs) have been proposed as a lightweight, cost-efficient, and ubiquitous solution. Importantly for IoT developers, PUFs promise to achieve perfectly secure authentication without any cryptographic assets on the device, which makes them especially interesting for resource-scarce IoT devices [11,12]. The characteristics of a PUF include unpredictable response (R) as an output of the system owing to intrinsic variations, which are stimulated by the input challenge (C). The challenge–response system is modeled as a black box; hence, the PUF input–output relation is described as R = f(C), where f(.) is an unknown internal parameter influenced by the intrinsic variations of the device [13,14]. Practically, no two chips generate identical responses for a particular challenge, as depicted in Figure 1. The combination of a challenge and its corresponding response is called a challenge response pair (CRP).

The PUF security metrics (the stability, the reliability, and the uniqueness) and the low power requirement have triggered enormous studies at the PUF cell level, such as investigations on the arbiter PUF [16,17,18], ring-oscillator (RO) PUF [19,20,21,22,23,24], be-stable ring PUF [25], glitch PUF [26], dynamic random access memory (DRAM) PUF [27,28], and static random access memory (SRAM) PUF [29,30]. In addition, many studies on low-power PUF circuits have been conducted, such as robust digital response and low-power current-based PUF [31], as well as the SRAM-based PUFs, over a wide range of supply voltages from the super-threshold voltage regime down to the near-threshold voltage (NTV) regime [32]. It has to be noted that the IoT devices are mostly battery-powered embedded devices and operate in low-frequency ranges. Meanwhile, all PUF cells presented in [16,17,18,19,20,21,22,23,24,25,26,27,28,29,30] undergo high-power consumption and voltage ramp-up time adaptation for a reliable PUF circuit [33]. To address for low-power and stable PUF security profiles, the low-power technique of the adiabatic switching principle [34] has been adopted in SRAM style PUF cell design [33,35].

Continuous scaling-down of planar MOSFETs over the past four decades has delivered ever-increasing transistor density into integrated circuits (ICs). However, continuing this trend in the nanometer regime is very challenging due to the drastic increase in the subthreshold leakage current (*I*off) [36,37,38]. Due to the very narrow channel lengths in deeply scaled MOSFETs, the drain potential begins to influence the electrostatics of the channel and, consequently, the gate loses adequate control over the channel. As a result, the gate is unable to shut off the channel completely in the off-mode of operation, which leads to an increased *I*off between the drain and the source [39]. Very limited work on employing FinFET-based PUF has reported, such as FinFET-based hybrid ring arbiter PUF [40] and oscillator collapse and time collapse comparator PUFs [41].

In this work, a novel study on FinFET-based SRAM PUF is presented. The previous work on two-phase clocking adiabatic PUF (TPCA-PUF) [33] cells is investigated using the dual-gate FinFET 45 nm process with 1.0 V nominal supply voltage. The uniqueness, reliability, and the BER of the proposed TPCA-PUF are compared with the QUALPUF [35] using the same process technology. 

The remainder of this paper is structured as follows: Section 2 introduces the FinFET-based adiabatic PUF by first explaining the fundamental adiabatic logic current trace compared to the traditional logic style, FinFET physical structures, the proposed FinFET adiabatic PUF, and its implementation into 4-bits LSI PUF. The simulation conditions, security evaluation metrics, and simulation results are described in Section 3. The evaluation of the proposed work in comparison with conventional PUFs is discussed in Section 4. Finally, Section 5 concludes the research findings of this work.

## 2. Adiabatic FinFET-Based PUF

### 2.1. Adiabatic Logic

**Figure 2:**
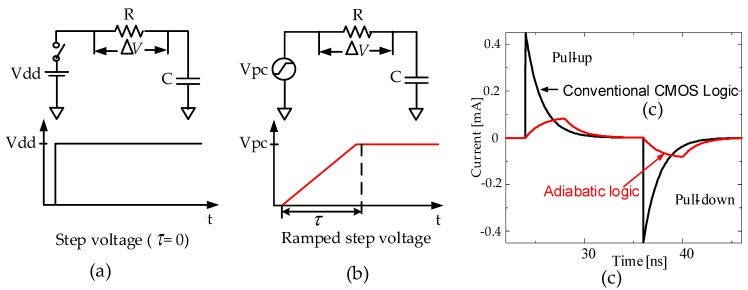
Comparison of the supply currents for the equivalent RC models: (**a**) CMOS logic with step voltage, (**b**) adiabatic logic with ramped step voltage, and (**c**) the peak supply current of the adiabatic logic is significantly lower than that of the conventional CMOS logic under the same parameters and conditions.

Adiabatic switching is commonly used for minimizing the energy lost during the charging/discharging period at all nodes of the circuit. The main concept of adiabatic switching is shown in Figure 2b, which indicates a transition that is considered sufficiently slow such that heat is not significantly emitted. The adiabatic dissipated energy is expressed as:

(1) $$E_{\mathrm{Adiabatic}}=\frac{RC}{\tau }C{V_{\mathrm{dd}}}^{2}$$

where *R* is the effective resistance in the driven device, *C* is the output node capacitance to be switched, *τ* is the time over which switching occurs, and V_dd_ is the voltage to be switched across. Ideally, the charging energy, E_Adiabatic_, tends to zero by increasing the length of *τ*. Conversely, the conventional CMOS logic operation is shown in Figure 2a, with the following equation:

(2) $$E_{\mathrm{CMOS}}=\frac{1}{2}C{V_{\mathrm{dd}}}^{2}$$

Here, in Equation (2), it is possible to reduce the charging energy only by reducing Vdd or capacitor, C. Figure 2c shows a comparison of the peak supply current for the equivalent RC models of the conventional CMOS logic versus the adiabatic logic. The comparison result in this figure shows that the instantaneous peak supply current of the adiabatic logic is significantly lower than that of the conventional CMOS logic style.

### 2.2. Fundamental of FinFET

**Figure 3:**
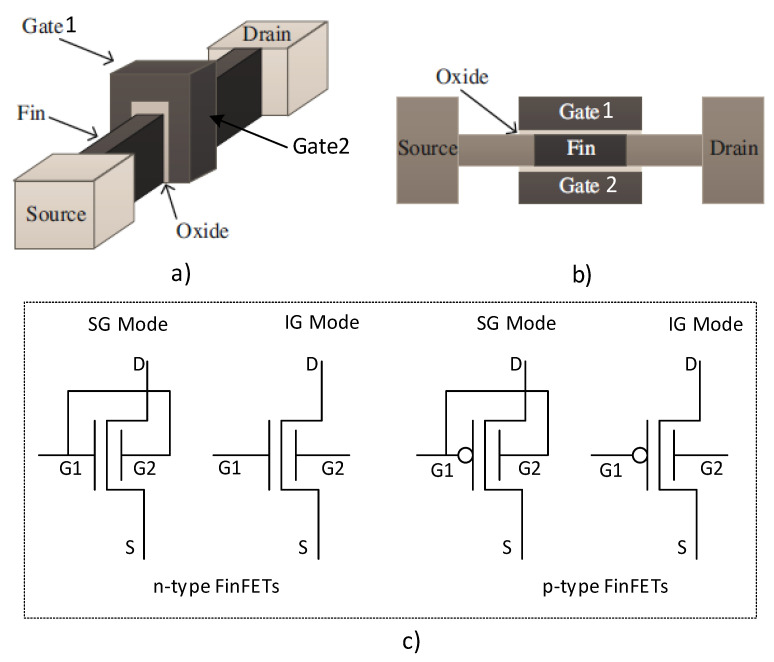
FinFET model and schematic: (**a**) three-dimensional structure, (**b**) cross-sectional top view, and (**c**) schematic symbol (modified from [42]).

With the scaling down of the transistor conductive channel, the short-channel effects (SCEs) become intolerable, and many multi-gate devices have been proposed to overcome the SCEs. Due to the relatively simple manufacturing process and the good compatibility with bulk CMOS, FinFET is considered to be a double gate (front gate and back gate) that can replace the high *k* of the planar MOSFET. The FinFET device has the advantages of higher on-state current, lower off-state current (lower leakage current), and faster switching speed [42]. The unique characteristic of the FinFET is that the conducting channel consists of thin silicon, known as “fin”, which forms the body of the device. Moreover, FinFET is also known as a non-planner double-gate MOSFET (DG-MOSFET), in which both the front gate (FG) and back gate (BG) are tied together. A typical FinFET structure is shown in Figure 3, in which Figure 3a,b depict the three-dimensional structure and cross-sectional top view of the FinFET, respectively. Figure 3c demonstrates the schematic symbol with shorted-gate (SG) mode and independent-gate (IG) mode of the n-type and p-type FinFETs. Furthermore, according to front gate and back gate being tied up or not, FinFET circuits can be divided into three different operating modes, namely, SG, IG, and the low-power gate (LP) modes [43].

### 2.3. Adiabatic FinFET TCPA-PUF

**Figure 4:**
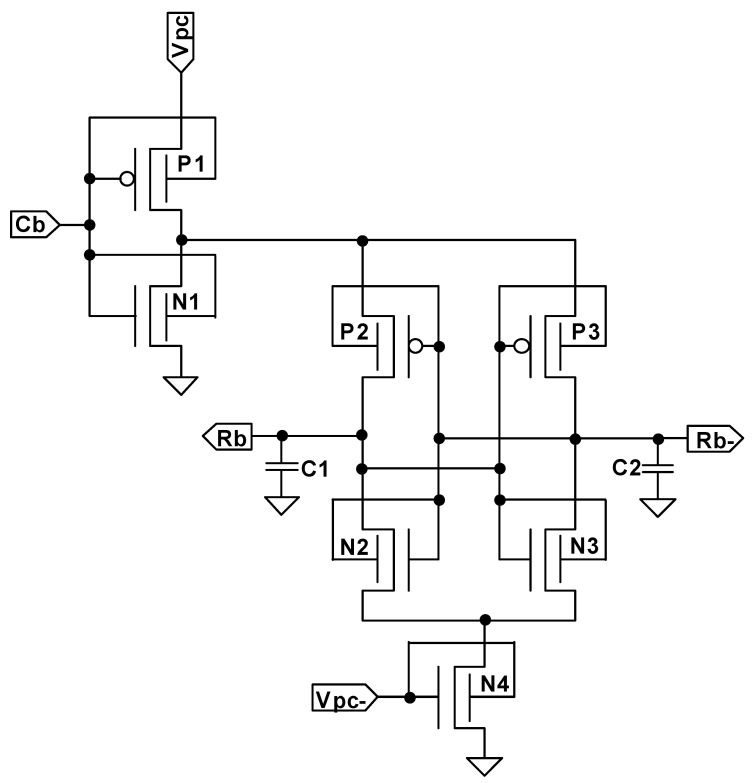
Proposed SRAM-based FinFET TPCA-PUF circuit.

The proposed FinFET-based TPCA-PUF circuit topology is shown in Figure 4. The CMOS-based TCPA-PUF was proposed in [33], which is adiabatically operated in four phases (wait, evaluate, hold, and recover phases). It consists of a static CMOS inverter (P1 and N1), which plays a key role in charging and discharging the PUF cell semi-adiabatically using a trapezoidal power clock signal of Vpc and controlled by the Cb. Similar to the QUALPUF circuit [35], the proposed TCPA-PUF cell consists of a cross-coupled inverter (P2, P3, N2, and N3) to evaluate the response-bits (output nodes of Rb and Rb- in Figure 4). As an improvement from the QUALPUF one, the current flow from one of the output nodes is controlled to slowly flow to the ground through transistor N4, by controlling its operation speed with a ramped Vpc- signal.

In this work, the same circuit topology is then further investigated using bulk-type FinFETs in SG mode to limit the leakage current that may flow through static inverter P1 and N1 transistors. To the best of our knowledge, this is the first work on SRAM-based FinFET PUF cells in adiabatic operation for ultra-low-power and high performance (security metrics) for secure key generation and/or other related weak PUF application.

### 2.4. 4-Bits Adiabatic FinFET TPCA-PUF

**Figure 5:**
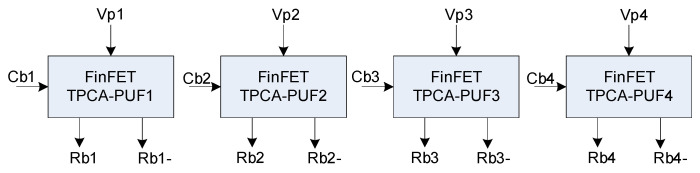
Proposed 4-bits FinFET TPCA-PUF architecture.

To validate the proposed FinFET TPCA-PUF cell, we have designed a 4-bits cascaded adiabatic PUF, as depicted in Figure 5. Each local PUF is supplied by 4 power clocks, where each adjacent clock differs by a phase difference of 90°. For instance, if the first cell, as shown in Figure 5, is operating in the hold phase, the next cell in the same local PUF is operating in the recovery phase. Similarly, the other two PUFs are operating in the wait phase and the evaluate phase, respectively. When all 4 outputs are sampled simultaneously, it leads to a 4-bit-length. Moreover, each local PUF cell is controlled by challenge bits, and each adjacent bit has a ¼ delay time of one power clock cycle. This means that if one cycle of the Vpc signal is 10 ns, then the second *Cb* signal has a 2.5 ns delay time compared to the first *Cb* signal. This delay time allows the challenge bits to flip the response signals right at the middle point of the idle/wait phase of the Vpc signals, and the challenge bits are perfectly flipped adiabatically, and as result of this, the energy is significantly reduced, as reported in [33].

**Figure 6:**
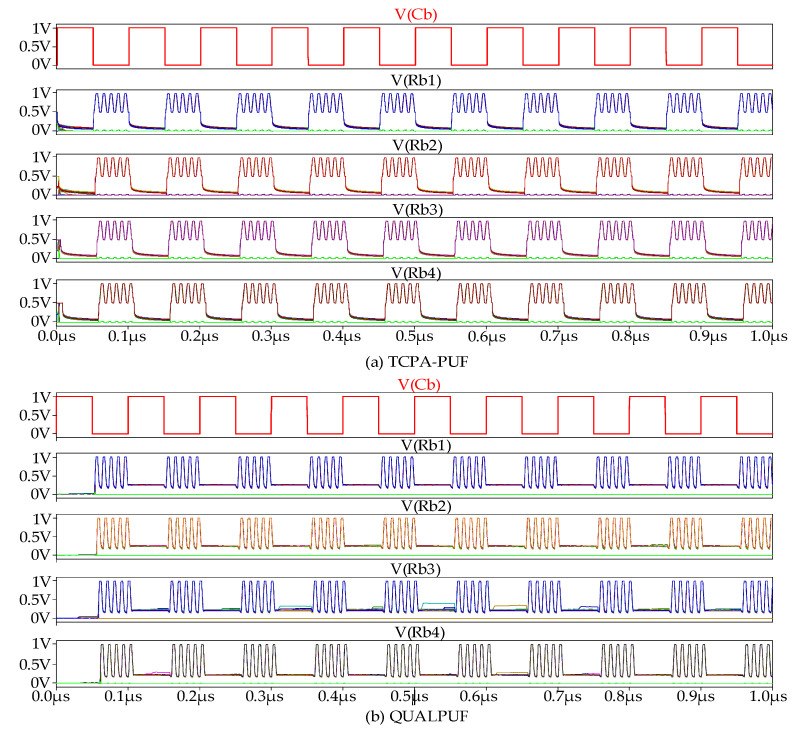
4-bits LSI circuit Monte-Carlo simulation result: (**a**) proposed FinFET-based TPCA-PUF and (**b**) FinFET-based QUALPUF.

Monte-Carlo simulation result of the 4-bits TPCA-PUF and QUALPUF challenge–response signals are depicted in Figure 6. This result was obtained with a reference temperature of T = 27 °C and C_L_ = 10 fF, *f_Cb_* = 10 MHz, and *f_Vpc_* = 100 MHz, with ±10% of Vth variation. The simulation results of response signals (Rb1–Rb4) with the given challenge bit (Rb) showed correct and stable operation for both PUF circuit topologies.

## 3. Simulation and Results

### 3.1. Simulation Condition

To analyze the effectiveness of the proposed FinFET-based TPCA-PUF, the 4-bit cascaded PUF was simulated using a 45 nm bulk FinFET standard process. Table 1 summarizes the device parameters and the simulation conditions.

For the ability of PUF to uniquely distinguish a chip among the group of other chips, 100 runs of the Monte-Carlo simulation were conducted to emulate the behavior of 100 IC PUF chips. This simulation aimed to analyze the effects of the process variation, such as threshold voltage (*V_TH_*) variation, the gate oxide thickness (*T_OX_*) variation, and the ambient temperature variation. The ±10% tolerance variation (for *V_TH_* and *T_OX_*) was set for the Monte-Carlo simulation condition to evaluate the uniqueness and the reliability of the proposed adiabatic PUF cell. Moreover, the ±20% of supply voltage was applied to further investigate the reliability of the proposed TPCA-PUF circuit. The temperature variation was set at −40, 0, 27, 50, and 100 °C.

### 3.2. Simulation Result

The relationship of input challenge bits and the output response bits of the proposed FinFET-based TPCA-PUF cell is in correct function, as depicted in Figure 6. For performance analysis, two major evaluation results are presented in this section: the energy dissipation per cycle per challenge bits (logic “1” and “0”), and the PUF evaluation metrics of uniqueness, reliability, and the bit-error-rate (BER).

### 3.3. Energy dissipation

**Figure 7:**
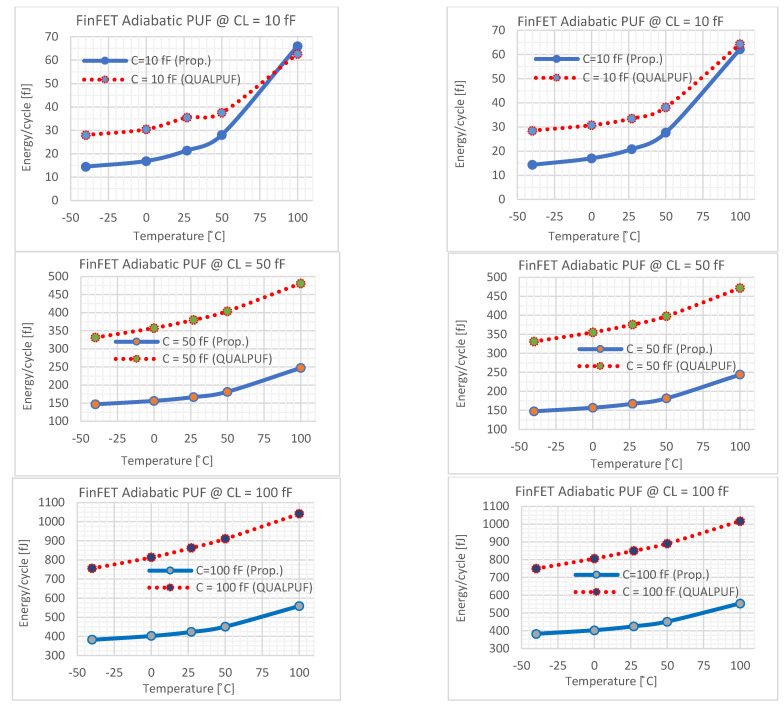

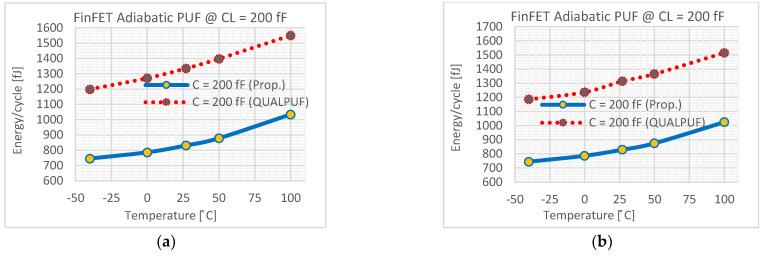
Energy consumption: (**a**) 10% of TOX variation and (**b**) 10% of V_TH_ variation, with load capacitance varying from 10 to 200 fF.

One of the major reasons for employing adiabatic switching techniques in PUF circuits is to reduce power consumption. In this study, the energy is collected from the total instantaneous power along the duration of the challenge bits cycle, as follows:

(3) $$E=\int_{0}^{T}\Sigma \left(VpcsIpcs\right)dt$$

where T signals the period of the challenge bits (*f_Cb_* = 10 MHz or inversely equal to 100 ns). Energy dissipation of 4-bits TCPA-PUF is depicted in Figure 7. Figure 7a,b depict an energy consumption comparison for ±10% tolerance variation of *T_OX_* and *V_TH_,* respectively, for a load capacitance that varies from 10 to 200 fF. The results show that the proposed FinFET-based TPCA-PUF consumes lower energy compared to the QUALPUF topology [35].

### 3.4. PUF Evaluation Metrics

We utilized three evaluation metrics to verify the proposed adiabatic PUF behavior.

(1) The Uniqueness: Used to determine the ability of a PUF to uniquely distinguish a chip among the other chips [35]. The ideal value of the uniqueness metric is 50%. The uniqueness is expressed as:
  (4) $$\mathrm{Uniquness}\left(\%\right)=\frac{2}{k\left(k-1\right)}\overset{k-1}{\underset{i=1}{\sum }}\overset{k}{\underset{j=i+1}{\sum }}\frac{HD\left(R_{i},\;R_{j}\right)}{n}\times 100$$
  where *HD(R_i_, R_j_)* represents the Hamming Distance of two different PUF instances’ responses, with *k* = number of chips and *n* = bit-length.
(2) The Reliability: Measures the reproducibility of the challenge–response pairs of a PUF instance with the varying environmental conditions, such as temperature and process variations. The reliability is mathematically expressed as:
  (5) $$\mathrm{Reliability}\left(\%\right)=100-\frac{1}{k}\overset{k}{\underset{i=1}{\sum }}\frac{HD\left(R_{i,\;}R_{i,j}\right)}{n}$$
  where *k* represents the total number of chips (*k* = 100 in this work), *n* is the number of PUF bits, and *HD(R_i_, R_i,j_)* is the Hamming Distance of *j*-th sampling of *R_i_.* The ideal value of the reliability is 100%.
(3) Bit-Error-Rate (BER): Indicates the error rate of the reliability of a PUF circuit. The ideal performance of the reliability is 100%. However, it is difficult to achieve in practice. BER is expressed as:
  BER(%) = 100 − Reliability(%)(6)

**Figure 8:**
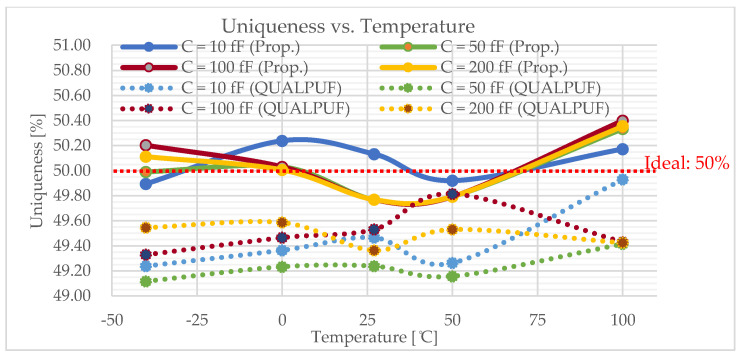
Uniqueness of the proposed FinFET-based TPCA-PUF (labeled as Prop.) versus QUALPUF under the V_TH_ variation (4-bit PUF).

**Figure 9:**
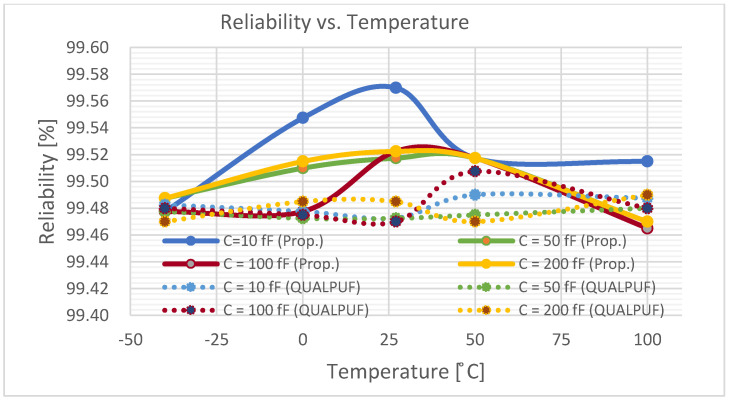
Reliability of the proposed FinFET-based TPCA-PUF (labeled as Prop.) versus QUALPUF under the V_TH_ variation (4-bit PUF).

**Figure 10:**
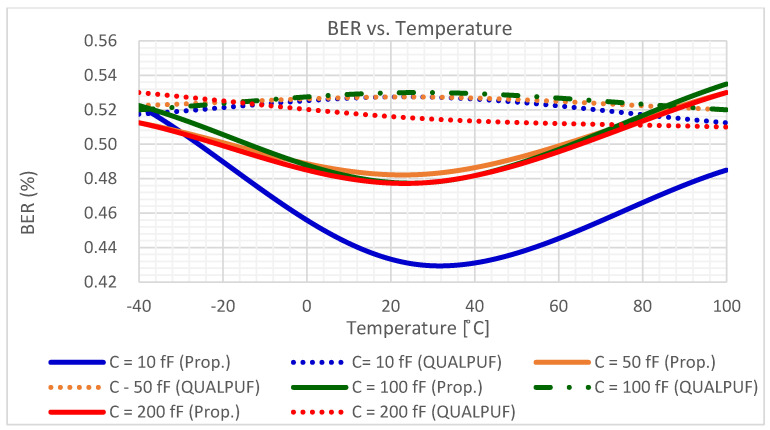
The BER of the proposed FinFET-based TPCA-PUF (labeled as Prop.) versus QUALPUF under the V_TH_ variation (4-bit PUF).

To enable the calculation of uniqueness, reliability, and the BER (Equations (2)–(4)), we conducted Monte-Carlo simulations with bit size of *n* = 4, and the chip number of *k* = 100. The calculation result of the uniqueness is graphically depicted in Figure 8, and the results of the reliability and the BER are shown in Figure 9 and Figure 10, respectively, all under the condition of ±10% *V_TH_* variations. Output nodes’ capacitors also vary from 10 to 200 fF to emulate the practical chip connection. In Figure 8, Figure 9 and Figure 10, the solid lines depict the proposed FinFET-based TPCA-PUF and the dotted lines show the FinFET-based QUALPUF.

## 4. Supply Voltage Variation

**Figure 11:**
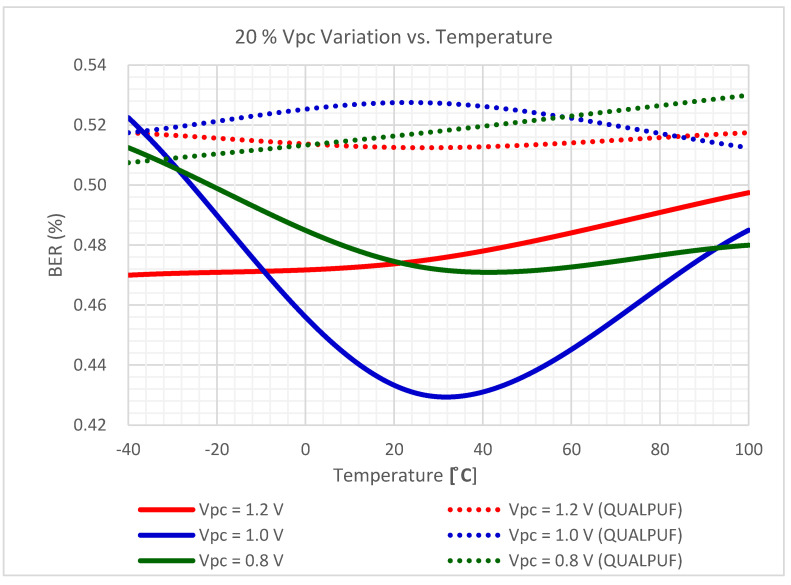
The BER of the proposed FinFET-based TPCA-PUF (labeled as Prop.) versus QUALPUF under the ±20% supply voltage variation (4-bit PUF).

Unexpected supply voltage fluctuation may also affect the reliability of any PUF performance. Hence, we have also further evaluated the reliability of the proposed adiabatic PUF against supply voltage variations. The 100 PUF instances were simulated under different supply voltages from 0.8, 1.0, and 1.2 V, for 45 nm bulked FinFET technology, and at three different temperatures of −40, 27, and 100 °C. The responses were read with 1.0 V as a reference, and then BER was calculated, as depicted in Figure 11. This figure indicates that the proposed TPCA-PUF cells are more resilient and stable under supply voltage variation at around normal temperatures.

## 5. Discussion

It has been revealed that the FinFET device has several advantages, such as higher on-state current, lower off-state current (lower leakage current), faster switching speed, and its double dual gates, enabling three possible connection modes (SG, IG, and LP) for low-power and high-speed applications. In this work, we have investigated the proposed TPCA-PUF cell using bulk FinFET with the 45 nm process for all SG, IG, and LP modes. As a result, we have found that SG mode is suitable for the proposed TPCA-PUF circuit topology. The gate connection type of LP and IG modes led to higher energy and produced the wrong response bits for a larger cascaded bit-length (4-bits in this work). Therefore, the whole work in this paper has utilized the SG mode connection type (refer to Figure 4).

The TPCA-PUF cell was implemented using SRAM-based circuit topology, and hence this study is claimed to be the first work in the literature employing FinFET-based SRAM-type PUF. Consequently, the previous reports of SRAM-based QUALPUF and TPCA-PUF [33,35] were re-simulated in the FinFET 45 nm process, as depicted in Figure 7, Figure 8, Figure 9, Figure 10 and Figure 11, and the key performances are described in Table 2. From Figure 7, Figure 8, Figure 9, Figure 10 and Figure 11, we can observe that the proposed FinFET-based TPCA-PUF demonstrated its superior performance (for both power and security metrics). Numerical data in Table 2 compare the QUALPUF and proposed TPCA-PUF, and more specifically, columns 4 and 6 demonstrate the simulation and calculation results from the current work (using the same parameters of the FinFET 45 nm process). Overall, data have shown that although FinFET-based QUALPUF and TPCA-PUF perform similar security metrics, the proposed TPCA-PUF consumes lower energy/bit/cycle and start-up power, which is suitable for low-power IoT applications.

## 6. Conclusions

In this study, we have presented an investigation study on SRAM-based FinFET PUF using the 45 nm technology process. The previous proposed TPCA-PUF was further investigated in a 4-bits cascaded bit-length, where the evaluation metrics of power consumption, the uniqueness, reliability, and the BER have been reported. For the low-power requirement, the proposed TPCA-PUF has reduced energy/bit/cycle and start-up power, both by about 70% compared to the QUALPUF cell at the same reference temperature of 27 °C.

The uniqueness, reliability, and the BER of the proposed FinFET-based TPCA-PUF were 50.13%, 99.57%, and 0.54%, which shows a superior security performance if compared with the FinFET-based QUALPUF cell. The remarkable performance (ultra-low power and security profile) of the proposed FinFET-based TPCA-PUF makes it an appropriate candidate for low-power and secure IoT device applications.

Further studies will be addressed to investigate the proposed TPCA-PUF with 128-bits, and address LSI implementations.

## Figures and Tables

**Table 1:** Simulation conditions.

**Power supply**	- Vpc: swing from 0.5 to 1.0 V trapezoidal clock, *f_Vpc_* = 100 MHz - Vpc-: swing from 0 to 0.5 V trapezoidal power clock, *f_Vpc-_* = 100 MHz - *Cb* voltage: 1.0 pulse signal, *f_Cb_* = 10 MHz
**Transistor parameter**	- 45 nm double-gate FinFET process

**Table 2:** Comparison of conventional and proposed adiabatic PUFs (with T = 27 °C and C_L_ = 10 fF, *f_Cb_* = 10 MHz, and *f_Vpc_* = 100 MHz).

PUF	QUALPUF [35]			TCPA-PUF	
	CMOS		FinFET	CMOS	FinFET
Year	2020		This Work	2021 [33]	This Work
Tech. Process (nm)	180 nm	45 nm	45 nm	180 nm	45 nm
Topology	Adiabatic SRAM			Adiabatic SRAM	
Transistor number/bit	5	5	5	7	7
Start-up power	3.08 µW	NA	65.69 nW	0.47 µW	18.32 nW
Energy (fJ/bit/cycle)	39.18	0.08	7.36	15.98	2.30
Uniqueness (%)	40.50	49.41	49.46	49.82	50.13
Reliability (%)	96.20	99.60	99.47	99.47	99.57
BER (%)	3.8	0.4	0.53	0.53	0.43

## Data Availability

Data available on request from the authors.
